# Engineering storage capacity for volatile sesquiterpenes in *Nicotiana benthamiana* leaves

**DOI:** 10.1111/pbi.12933

**Published:** 2018-05-28

**Authors:** Thierry L. Delatte, Giulia Scaiola, Jamil Molenaar, Katyuce de Sousa Farias, Leticia Alves Gomes Albertti, Jacqueline Busscher, Francel Verstappen, Carlos Carollo, Harro Bouwmeester, Jules Beekwilder

**Affiliations:** ^1^ Lab Plant Physiol Wageningen Univ & Res Wageningen The Netherlands; ^2^ Lab Prod Nat & Espectrometria Massas Univ Fed Mato Grosso do Sul Campo Grande MS Brazil; ^3^ Lab Ecologia & Espectrometria Massas Univ Fed Mato Grosso do Sul Campo Grande MS Brazil; ^4^ Wageningen Univ & Res Wageningen Plant Res Biosci Wageningen The Netherlands; ^5^ Present address: Swammerdam Institute for Life Sciences University of Amsterdam Amsterdam The Netherlands

**Keywords:** lipid bodies, triacylglycerol, secondary metabolites, sesquiterpene, *Nicotiana benthamiana*, storage

## Abstract

Plants store volatile compounds in specialized organs. The properties of these storage organs prevent precarious evaporation and protect neighbouring tissues from cytotoxicity. Metabolic engineering of plants is often carried out in tissues such as leaf mesophyll cells, which are abundant and easily accessible by engineering tools. However, these tissues are not suitable for the storage of volatile and hydrophobic compound such as sesquiterpenes and engineered volatiles are often lost into the headspace. In this study, we show that the seeds of *Arabidopsis thaliana*, which naturally contain lipid bodies, accumulate sesquiterpenes upon engineered expression. Subsequently, storage of volatile sesquiterpenes was achieved in *Nicotiana benthamiana* leaf tissue, by introducing oleosin‐coated lipid bodies through metabolic engineering. Hereto, different combinations of genes encoding diacylglycerol acyltransferases (DGATs), transcription factors (WRINKL1) and oleosins (OLE1), from the oil seed‐producing species castor bean (*Ricinus communis*) and Arabidopsis, were assessed for their suitability to promote lipid body formation. Co‐expression of α‐bisabolol synthase with Arabidopsis *DGAT1* and *WRINKL1* and *OLE1* from castor bean promoted storage of α‐bisabolol in *N. benthamiana* mesophyll tissue more than 17‐fold. A clear correlation was found between neutral lipids and storage of sesquiterpenes, using synthases for α‐bisabolol, (*E*)‐β‐caryophyllene and α‐barbatene. The co‐localization of neutral lipids and α‐bisabolol was shown using microscopy. This work demonstrates that lipid bodies can be used as intracellular storage compartment for hydrophobic sesquiterpenes, also in the vegetative parts of plants, creating the possibility to improve yields of metabolic engineering strategies in plants.

## Introduction

Sesquiterpenes form a structurally diverse family of hydrophobic molecules. They consist of three isoprene units concatenated by the farnesyl diphosphate synthase in to the sesquiterpene precursor farnesyl diphosphate (FPP). The sesquiterpene synthase enzymes are functioning via a carbocation rearrangement, and their structural diversity is further increased by oxidation (Vickers *et al*., 2014). Plants produce sesquiterpenes as bioactive compounds to protect themselves from insects and pathogenic microorganisms. Plant‐produced sesquiterpenes are used as ingredients in pharmaceutical, cosmetic and flavouring products. The commercial exploitation of natural terpene‐containing plant sources requires sustainable plant cultivation. This is particularly challenging when the source plant accumulates the compound of interest in economically viable quantities only after a growth period of 10–15 years. Unregulated collection of such plants may endanger their wild populations and ecosystems. For example, the wood of the Candeia tree (*Eremanthus erythropappus* (DC.) McLeish), which grows in Brazilian semi‐arid regions, has been used extensively as a source of α‐bisabolol (de Padua *et al*., 2016). The steam‐distilled wood oil, containing 85% α‐bisabolol, is used in pharmaceutical and cosmetic skin care products for its wound healing properties (Kamatou and Viljoen, 2010). As a consequence of overexploitation of the naturally occurring populations, Candeia material is no longer sufficiently available to meet market demands for α‐bisabolol, and alternative sources for this compound are under investigation (Han *et al*., 2016; de Meireles *et al*., 2015; Son *et al*., 2014).

Metabolic engineering could provide alternative sources for important sesquiterpenes such as α‐bisabolol. Studies into the heterologous expression of terpene synthases in fast‐growing plant species such as *N. benthamiana* have provided strategies for overproduction of sesquiterpenes in leaf mesophyll cells. Changing the targeting of terpene synthases from the cytosol to plastids or mitochondria has been shown to enhance sesquiterpene production (van Herpen *et al*., 2010; Wu *et al*., 2012). Overexpression of enzymes supplying FPP, the immediate precursor of sesquiterpenes, also resulted in enhanced terpene production (van Herpen *et al*., 2010; Wu *et al*., 2012). Down‐regulating expression of pathways that compete for FPP, for example phytosterol biosynthesis, also enhanced heterologous terpene production (Cankar *et al*., 2015; Chen *et al*., 2000). However, the capacity of the mesophyll cells for sequestering the overproduced terpenes is limited. As a consequence, enhanced production often leads to enhanced emission, rather than higher accumulation of the sesquiterpene (van Herpen *et al*., 2010).

Plants that are naturally rich in hydrophobic compounds such as sesquiterpenes have evolved different strategies to prevent their evaporation under ambient temperatures in aqueous environments (Keating *et al*., 2017; Widhalm *et al*., 2015). Often, sesquiterpenes are stored in specialized tissues, to isolate them from largely aqueous mesophyll tissues where photosynthesis is taking place. As a result, their evaporation is delayed, and their cytotoxicity is contained, until they are needed for the defence of the plant against insects or microbes. Well‐studied examples of such tissues include glandular trichomes, idioblasts and resin ducts (Geng *et al*., 2012; Lange, 2015; Mewalal *et al*., 2017; Tissier *et al*., 2017). All these plant organs provide a core of hydrophobic material where terpenes can accumulate, which is isolated from the surrounding tissue by a protective layer. Upon tissue damage, for example by pest attack, but also by extraction procedures such as steam distillation, the protective layer is broken and the sesquiterpenes are released.

Plants also contain lipid bodies, which can also be considered as contained hydrophobic environments. Lipid bodies are hydrophobic particles that predominantly occur in oil‐rich seeds, for instance those of Arabidopsis, sunflower and castor bean (Branham *et al*., 2016; Chen *et al*., 2007; Gao *et al*., 2017). Lipid bodies consist of triacylglycerols (TAG), which assemble into particles of defined size and are clattered by small proteins called oleosins (Pyc *et al*., 2017). The cellular processes required for formation of lipid bodies are largely understood from work in Arabidopsis. A number of transcription factors, among which are WRINKL1 and LEC2, up‐regulate the biosynthesis of fatty acids (Vanhercke *et al*., 2017). Through the activity of a diacylglycerol acyltransferase (DGAT), these are condensed into TAG molecules that are deposited between the two phospholipid monolayers of the ER membrane (Chapman *et al*., 2012). By action of oleosins, the TAG deposits bud away from the ER membrane and form intracellular hydrophobic particles (Napier *et al*., 1996). These particles have been found in lipid‐accumulating storage organs such as seeds. However, there is a growing interest in increasing the TAG content of vegetative tissues, as a carbon neutral alternative to petroleum (Carlsson *et al*., 2011). Therefore, engineering tools have been developed that could mediate lipid body formation in tissues other than seeds. A core set of genes required for the formation of lipid bodies has been identified (Vanhercke *et al*., 2013, 2014, 2017; Xu and Shanklin, 2016; Zale *et al*., 2016).

In this work, we explore the possibility to trap volatile sesquiterpenes inside lipid bodies *in vivo*. The concept of using lipid bodies as storage of sesquiterpenes *in planta* is demonstrated by co‐engineering sesquiterpene biosynthesis and lipid body formation in leaf mesophyll tissue in *N. benthamiana* plants.

## Results

### Seeds of *35S: AtTPS21* expressing Arabidopsis trap (*E*)‐β‐caryophyllene

This research was initiated when a construct expressing the Arabidopsis (*E*)‐β‐caryophyllene synthase *AtTPS21* using the 35S promoter was introduced in Arabidopsis *thaliana* (Columbia 0), and seeds from confirmed transformed plants were analysed by GC‐MS (Ting *et al*., 2015). The dry seeds of the 35S:*AtTPS21* plants contained a significant level of (*E*)‐β‐caryophyllene, whereas the WT seeds did not. This result is in line with similar work in *Camelia sativa*, where plants expressing terpene synthases accumulated terpenes in seed oils (Augustin *et al*., 2015; Borghi and Xie, 2016).

**Figure 1:**
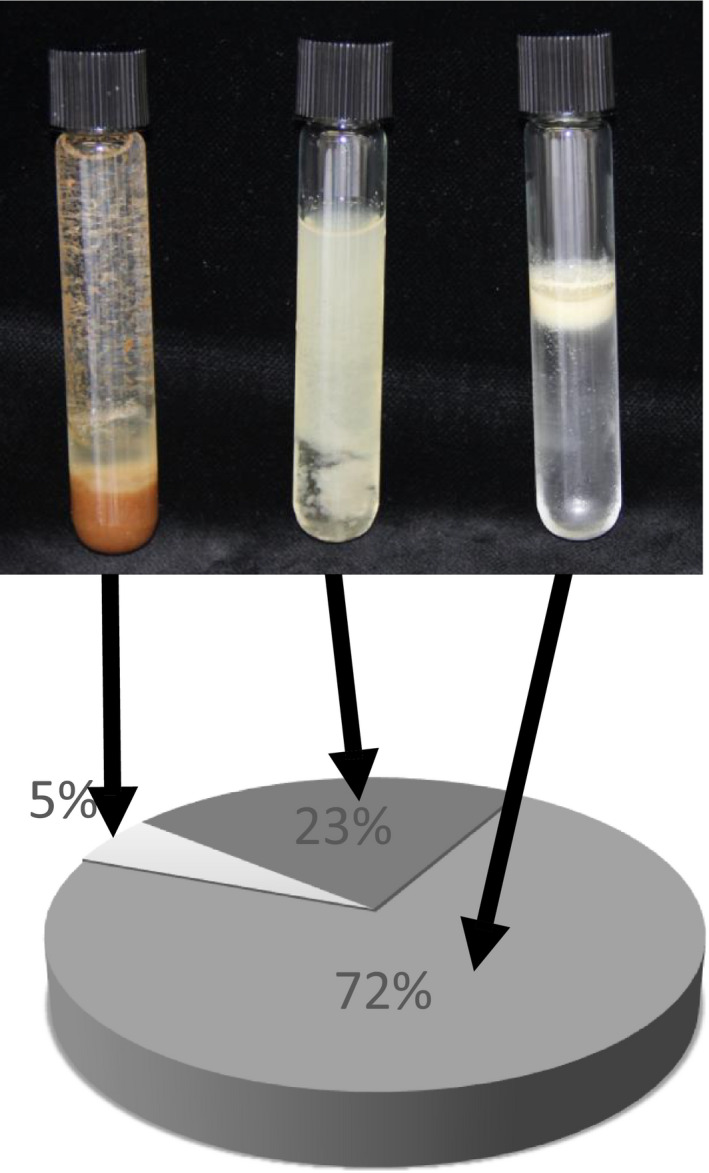
Sesquiterpene content of seed fractions. Arabidopsis *35S:AtTPS21* seeds were homogenized and separated on a sucrose gradient resulting in three distinct fractions: insoluble (upper panel left), soluble (upper panel middle) and fat layer (upper panel right). These three fractions were then analysed by GC‐MS after hydrodistillation to quantify the amount of (*E*)‐β‐caryophyllene present (lower panel). Each slice is the average of three independent measurements.

To further investigate the localization of *(E*)‐β‐caryophyllene in the *35S:AtTPS21* Arabidopsis seeds, material was ground and subjected to sucrose gradient centrifugation. In this gradient, lipid bodies will migrate to the top to form an upper layer (Ding *et al*., 2013). Three resulting fractions (pellet, soluble and upper layer) were hydrodistilled and analysed by GC‐MS (Figure 1). Most of the (*E*)‐β‐caryophyllene (72% ± 4, *n* = 3) was found in the upper layer, co‐localizing with the lipid bodies. The lipid content of Arabidopsis seeds of the Columbia 0 ecotype is 35% (w/w) (Li *et al*., 2006). This suggests that lipid bodies naturally present in plant tissues can function as storage for sesquiterpenes.

### Increase in total lipids in *N. benthamiana* mesophyll upon transient expression of *OLE1* and *DGAT1*

**Figure 2:**
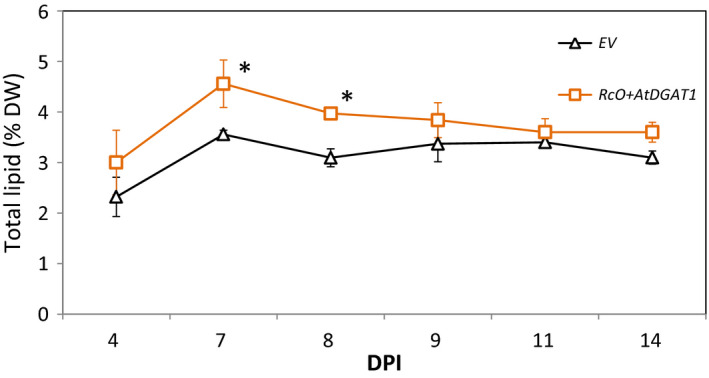
Lipid content over time in agro‐infiltrated leaf from *N. benthamiana*. The optimal sampling point for the rest of the study was identified by plotting the total lipid content, analysed by gravimetry, of leaves agro‐infiltrated with *RcO + AtDGAT1* (squares) and empty‐vector control (EV, triangles) over a period of 10 days starting at 4 DPI. Each point is the average of five biological replicates ±SE, a start indicates the significant difference between the two treatments at a given time point. DW, dry weight; DPI, day post‐agro‐infiltration; EV, empty binary vector; *RcO, Ricinus communis OLEOSIN; AtDGAT1, Arabidopsis thaliana* diacylglycerol transferase.

Transient expression using agro‐infiltration of *N. benthamiana* was deployed to engineer formation of lipid bodies in leaf mesophyll cells. Constructs containing *DGAT1* from Arabidopsis (*AtDGAT1*) and *OLE1* from castor bean (*RcO*) driven by a 35S promoter were generated, individually transformed to *A. tumefaciens,* and subsequently co‐infiltrated into *N*. *benthamiana*. This combination is known to increase the TAG level in vegetative tissues after agro‐infiltration (Vanhercke *et al*., 2013). To determine the time at which the lipid content due to expression of these genes is the highest, agro‐infiltrated leaves were harvested at six time points: from 4 to 14 DPI (day post‐agro‐infiltration) (Figure 2). At 7 DPI and 9 DPI, total lipid content (on a dry weight basis) was significantly higher (*n* = 5, *P* < 0.05) in plants expressing *RcO + AtDGAT1*, relative to empty‐vector (EV) control plants. After 7 DPI, a decrease in total lipids in the *RcO + AtDGAT1* combination was observed, possibly indicating degradation of lipid bodies. Therefore, for further optimizations, 7 DPI was chosen as the preferred sampling time.

### Improving total lipid content

**Figure 3:**
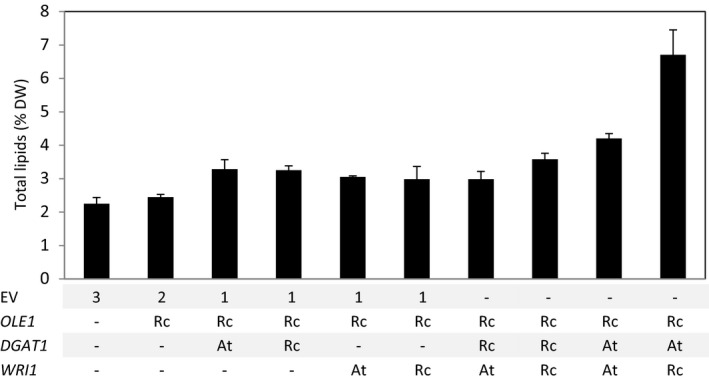
Total lipid content influenced by gene combinations. Total lipid content of agro‐infiltrated *N*. *benthamiana* leaves harvested at 7 DPI was gravimetrically analysed. Combinations of *OLE1*, *DGAT1* and *WRI1* originating from castor bean (Rc) or from *Arabidopsis thaliana* (At) were tested. Each bar represents the average of five biological replicates ±SE. DW, dry weight; EV, empty binary vector; OLE1, OLEOSIN; DGAT1, diacylglycerol transferase; WRI1, WRINKL1; Rc, *Ricinus communis*; At, *Arabidopsis thaliana*.

**Figure 4:**
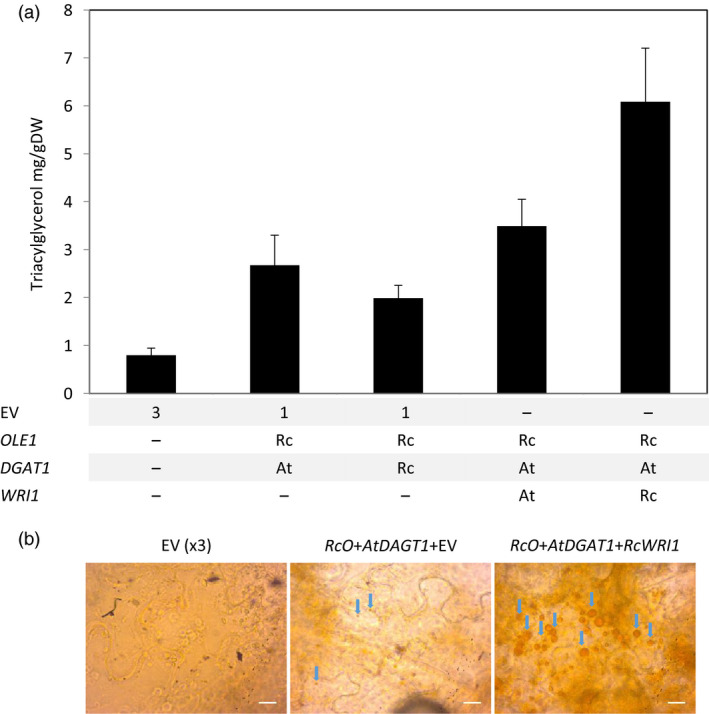
Transient expression of *RcO*, *DGAT1* and *WRI1* increases triacylglycerol content in *N. benthamiana leaves*. (a) Increased triacylglycerol content in leaf tissue at 7 DPI. The TGA content was quantified by TLC (bars are the average of five samples ±SE). (b) Light microscopy images of *N*. *benthamiana* leaves (at 7 DPI) after Sudan IV staining. Blue arrows indicate lipid bodies; bars correspond to 20 μm. EV, empty binary vector; Rc, *Ricinus communis*; At, *Arabidopsis thaliana*; OLE1, OLEOSIN; DGAT1, diacylglycerol transferase; WRI1, WRINKL1; RcO, *Ricinus communis* OLE1; AtDGAT1, *Arabidopsis thaliana* diacylglycerol transferase; RcWRI1, *Ricinus communis* WRINKL1.

To clearly demonstrate the potential of lipid bodies as a storage for terpenes in vegetative tissues, the ±20% increase in lipid content obtained by the expression of *RcO + AtDGAT1* was considered not sufficient. To further increase the amount of lipids, the seed filling transcription factor WRINKL1 (WRI1) was added to the system. WRI1 is known to boost fatty acid biosynthesis (An *et al*., 2017; Focks and Benning, 1998; Grimberg *et al*., 2015; Ma *et al*., 2013; Vanhercke *et al*., 2014). Additional constructs expressing *WRI1* from Arabidopsis (*AtWRI1*) and *WRI1* and *DGAT1* from castor bean (*RcWRI1* and *RcDGAT1*) were tested in a combinatorial approach, by agro‐infiltration in different combinations with *RcO*, and the infiltrated leaves analysed for total lipid content at 7 DPI (Figure 3). Two combinations clearly stood out from the others. *AtWRI1 + AtDGAT1 + RcO* yielded 4.2% (±0.14%) lipid (DW) and *RcWRI1 + AtDGAT1 + RcO* even 6.7% (±0.74%) (DW), while EV control leaves were significantly (*n* = 5, *P* < 0.05) lower in lipid content (2.2 ± 0.18% of DW). Apparently synthesis of TAGs in the presence of *AtDGAT1* is strongly stimulated by the castor bean *WRI1* transcription factor. Total lipids, as analysed above (Figure 3), represent not only TAGs, but also membrane phospholipids and other hydrophobic molecules. To confirm that the increased lipid content was the result of TAG accumulation, a subset of the constructs was agro‐infiltrated again and analysed by thin‐layer chromatography (TLC, Figure S1) for quantification of TAGs (Figure 4a). Overall, the differences observed in the TAG content confirmed the observation for the total lipid continent (Figure 3). Again, the highest content of TAGs was found in the *RcO + AtDGAT1 + RcWRI1* combination, where the quantity of TAG was 10‐fold higher than the EV control.

Mesophyll tissues were studied with light microscopy to observe morphological changes that accompany the increase in TAG content (Figure 4b and Figure S2), by staining with Sudan IV, a dye specific for neutral lipids (Andrianov *et al*., 2010; Bouvier‐Nave *et al*., 2000). While in EV control leaves, hardly any staining was observed, staining particles of diameters up to 6 μm with an average diameter of 2.8 ± 0.4 μm were observed in leaves where *AtDGAT1* and *RcO* were expressed. Larger and more numerous staining particles, of diameters up to 20 μm, with an average of 6.1 ± 0.7 μm, were observed in leaves expressing the *RcO* + *AtDGAT1 *+* RcWRI1* combination.

Thus, by transiently co‐expressing *RcO*,* AtDGAT1* and *RcWRI1* simultaneously in the mesophyll of *N. benthamiana*, a strong increase in TAG content was observed, as well as formation of lipid bodies. This combination of genes will be referred to as ODW from here onward.

### Lipid bodies promote α‐bisabolol storage

Having established a system for producing well‐detectable amounts of lipid bodies in *N. benthamiana* mesophyll cells, experiments were performed to address changes in the *in vivo* storage capacity for sesquiterpenes, due to the presence of lipid bodies. To this end, the *Artemisia annua* α‐bisabolol‐synthase cDNA (Li *et al*., 2013) was subcloned for plant expression in the pB7wG2.0 vector (*AaBOS*). Subsequently, *AaBOS* was co‐expressed with and without the ODW combination in *N. benthamiana* leaves, and accumulation of both TAGs and α‐bisabolol was analysed during 13 DPI, using TLC and GC‐MS, respectively.

**Figure 5:**
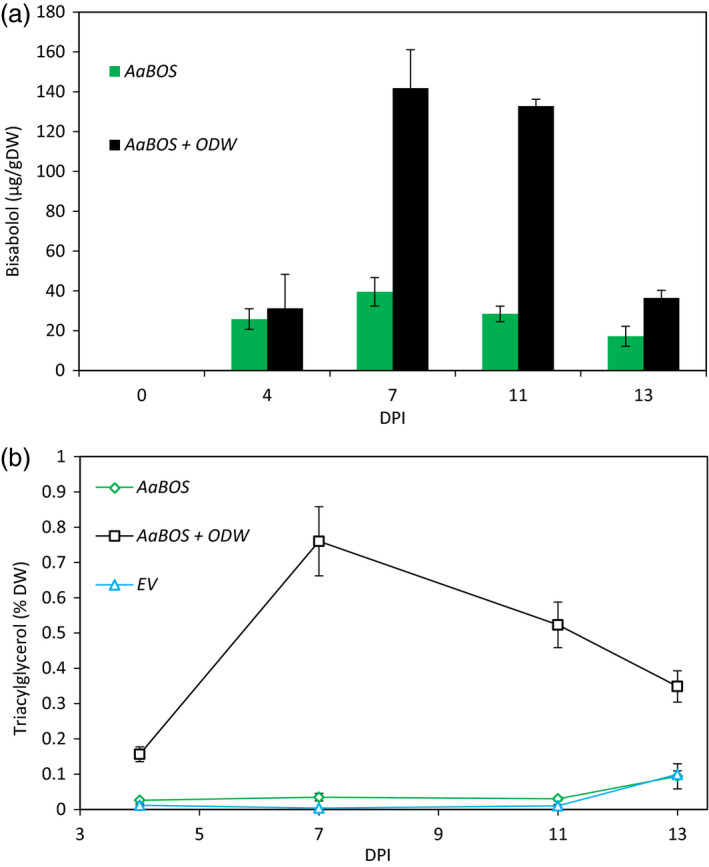
α‐Bisabolol storage and TGA accumulation in *N*. *benthamiana* leaves. (a) α‐Bisabolol content over time in *AaBOS* (dark grey) and *AaBOS *+ *ODW* (light grey) agro‐infiltrated *N. benthamiana* leaves. α‐Bisabolol was measured by GC‐MS and quantified by external calibration (bars are the average of five samples ±SE). (b) Triacylglycerol content over time in EV (dark grey dashed line), *AaBOS* (black solid line) and *AaBOS *+ *ODW* (light grey solid line) agro‐infiltrated *N. benthamiana* leaves. Triacylglycerol levels were measured by TLC and expressed in % of dry weight (each data point is the average of 5 samples ±SE). Samples were harvested at 4,7,11 and 13 DPI. DW, dry weight; DPI, day post‐agro‐infiltration; EV, empty binary vector; AaBOS,* Artemisia annua* bisabolol synthase; ODW,* Ricinus communis* OLEOSIN + *Arabidopsis thaliana* diacylglycerol transferase + *Ricinus communis* WRKINL1.

At 7 and 11 DPI, about fourfold more α‐bisabolol was detected in the leaves expressing the *ODW* combination than in leaves expressing only *AaBOS* (Figure 5a). At these time points, also the TAG content in the *ODW*‐expressing leaves was highest (Figure 5b and Figure S2). When TAG content decreased, at 13 DPI, α‐bisabolol content also decreased. Interestingly, the α‐bisabolol content did not fully parallel the accumulation of TAGs. At 4 DPI, where TAG content had already strongly increased, α‐bisabolol content had hardly increased in the *ODW*‐expressing leaves, compared to the EV control. At 11 DPI, when TAG content had already declined by ~40%, α‐bisabolol content was still very high.

### Is α‐bisabolol stored in the lipid bodies?

**Figure 6:**
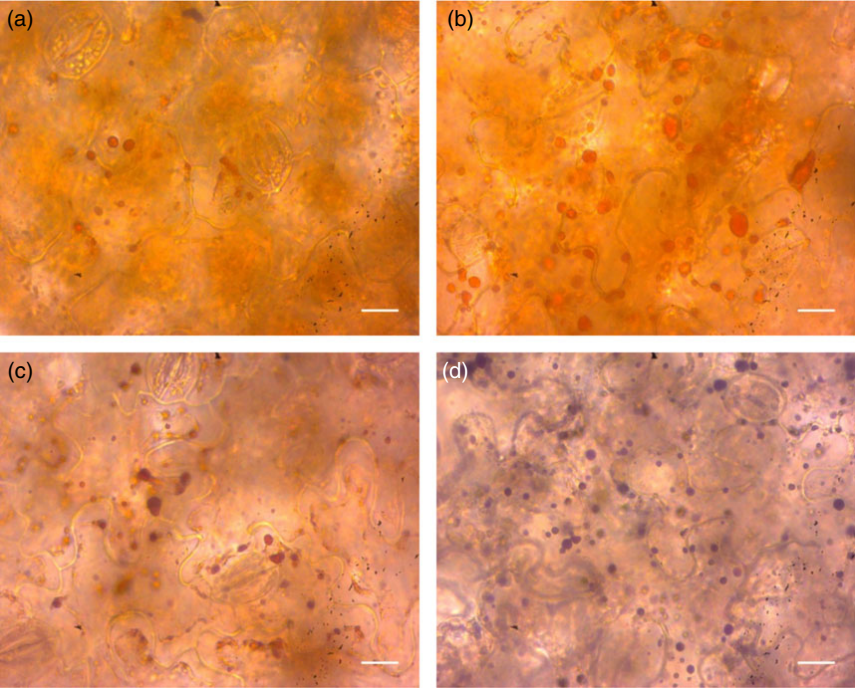
Histological localization of lipids and terpenes. (a) Tissue of EV + *ODW* stained with Sudan IV; (b) *AaBOS *+ *ODW* stained with Sudan IV; (c) EV + ODW stained with Sudan IV followed by a NADI staining; (d) *AaBOS *+ *ODW* stained with Sudan IV followed by a NADI staining. Samples were collected at 7 DPI, bars correspond to 20 μm. DPI, day post‐agro‐infiltration; EV, empty binary vector; AaBOS,* Artemisia annua* bisabolol synthase; ODW,* Ricinus communis* OLEOSIN + *Arabidopsis thaliana* diacylglycerol transferase + *Ricinus communis* WRINKL1.

To confirm that α‐bisabolol localizes to the lipid bodies *in vivo*, light microscopy was performed. The presence of lipid bodies was confirmed again by staining with the neutral lipid‐specific dye Sudan IV, which provides an orange colour to lipid bodies. No differences between tissues expressing *AaBOS *+ *ODW* (Figure 6a) and EV + *ODW* (Figure 6b) combinations could be observed. The same tissues were subsequently stained using the naphthol and diamine (NADI) reaction, which has been developed to selectively stain terpenes (David and Carde, 1964; Kolb and Muller, 2004). Here, the *AaBOS *+ *ODW* displayed purple staining of lipid bodies (Figure 6d), while, in the EV + *ODW* combination, these structures remained orange after the NADI staining (Figure 6c). The overlap of neutral‐lipid staining and terpene reactivity indicates that α‐bisabolol is stored in the lipid bodies.

### Higher α‐bisabolol production leads to higher trapping

**Figure 7:**
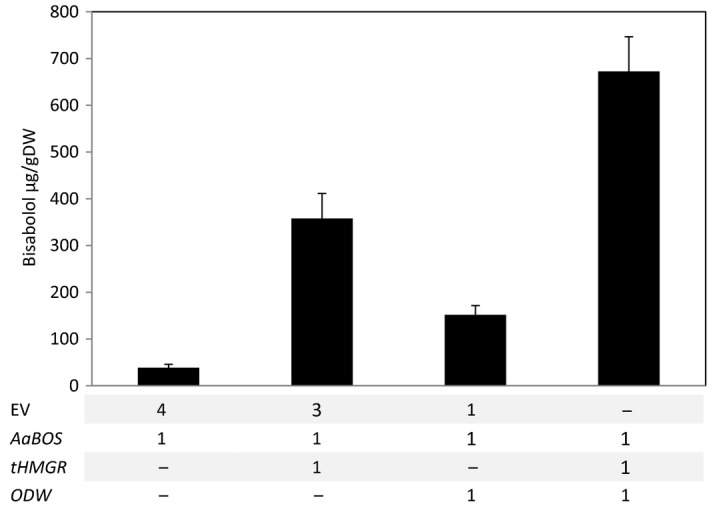
Enhancing the MVA pathway increases bisabolol storage in leaves. Bisabolol storage in *N. benthamiana* leaves was analysed after transient expression of: *AaBOS*,* AaBOS *+ *tHMGR* Aa *BOS *+ *ODW* and *AaBOS *+ *tHMGR *+ *ODW*. Bisabolol content was quantified by GC‐MS analysis on leaf extracts collected at 7 day post‐agro‐infiltration and freeze‐dried (each bar is the average of five biological replicates ±SE). DW, dry weight; EV, empty binary vector; AaBOS,* Artemisia annua* bisabolol synthase; tHMGR, truncated Arabidopsis thaliana 3‐hydroxyl‐3‐methyl‐glutaryl‐coenzyme A reductase; ODW,* Ricinus communis* OLEOSIN + *Arabidopsis thaliana* diacylglycerol transferase + *Ricinus communis* WRKINL1.

To test the limit of storage in these lipid bodies, production of α‐bisabolol was increased by enhancing the availability of its precursor α‐bisabolol. To this end, a truncated form of the 3‐hydroxy‐3‐methyl‐glutaryl‐coenzyme A reductase (*tHMGR*) was co‐expressed. The tHMGR is a deregulated version of the rate‐limiting enzyme in the mevalonate pathway, which supplies farnesyl diphosphate, the immediate precursor of sesquiterpenes such as α‐bisabolol (Cankar *et al*., 2015; van Herpen *et al*., 2010; Hey *et al*., 2006). Co‐expression of *AaBOS* with *tHMGR* leads to a 10‐fold increase in α‐bisabolol in the leaves (Figure 7). Combining *AaBOS* expression with both the *ODW* combination and *tHMGR* further increased the amount of α‐bisabolol stored in the lipid bodies, to about 20‐fold, compared to expression of *AaBOS* alone (Figure 7). The quantity of α‐bisabolol stored in leaves at 7 DPI was consistent between the independent experiments (Figures 5a and 7). Apparently, producing higher levels of α‐bisabolol results in an increase in α‐bisabolol storage in lipid bodies.

### (*E*)‐β‐caryophyllene and α‐barbatene are stored in lipid bodies

**Figure 8:**
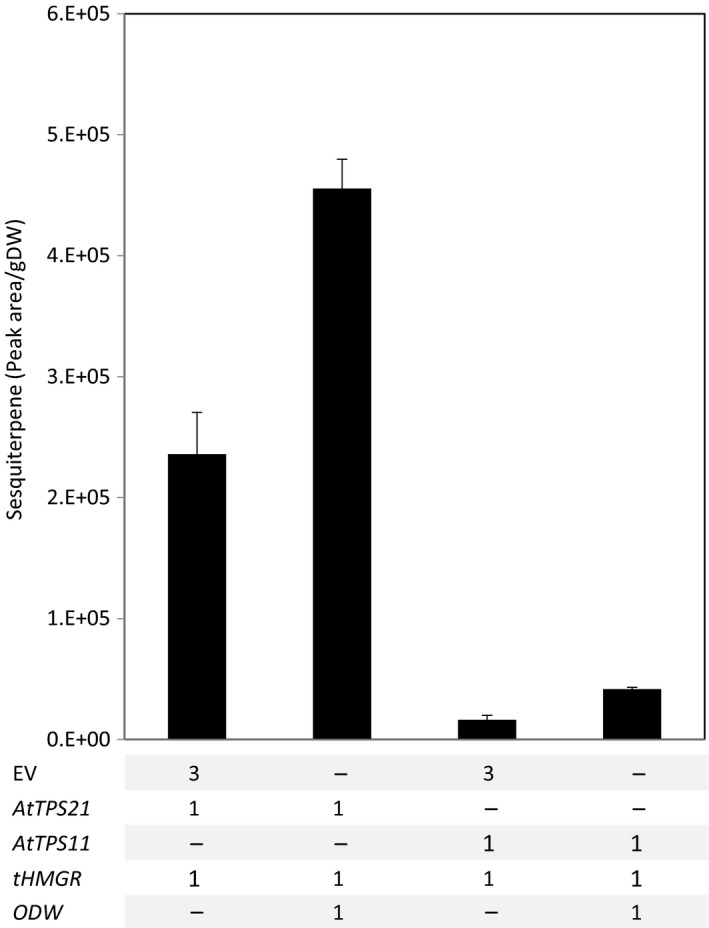
Improved storage for a broader sesquiterpene range. *AtTPS21* and *AtTPS11* in the presence of *tHMGR* were agro‐infiltrated with and without the *ODW* combination. After freeze‐drying, the tissue was extracted and analysed by GC–MS. The peak area of the synthase major product was selected. Each bar is the average of four replicate (±SE). DW, dry weight; EV, empty binary vector; AtTPS,* Arabidopsis thaliana* terpene synthase; tHMGR, truncated Arabidopsis thaliana 3‐hydroxyl‐3‐methyl‐glutaryl‐coenzyme A reductase; ODW,* Ricinus communis* OLEOSIN + *Arabidopsis thaliana* diacylglycerol transferase + *Ricinus communis* WRINKL1.

To investigate the storability of sesquiterpenes other than α‐bisabolol in the ODW‐induced lipid bodies, constructs for Arabidopsis terpene synthases *AtTPS11* and *AtTPS21* (Tholl *et al*., 2005) were generated. These two sesquiterpene synthase are responsible for most of the terpenes emitted by Arabidopsis flowers. AtTPS21 mediates biosynthesis of (*E*)‐β‐caryophyllene and AtTPS11 produces predominantly α‐barbatene (Tholl *et al*., 2005). Both *AtPS21* and *AtTPS11* were co‐expressed with *tHMGR* in the presence or absence of the ODW combination. When the ODW combination was present, a twofold increase in sesquiterpene storage for both sesquiterpene was observed (Figure 8).

## Discussion

The storage of volatile molecules has been a serious limitation to the bioengineering of monoterpenes and sesquiterpenes in plants and microorganisms. In this work, we present a novel strategy to store volatile sesquiterpenes in vegetative and reproductive plant tissues. This work was inspired by our finding that Arabidopsis overexpressing a (*E*)‐β‐caryophyllene synthase accumulates a significant amount of (*E*)‐β‐caryophyllene in seeds containing lipid bodies (Figure 1).

### Heterologously expressed lipid bodies enhance terpene storage

Increased TAG accumulation in *N. benthamiana* correlated with accumulation of α‐bisabolol (Figures 5, 6 and 7) and other terpenes (Figure 8). A twofold to fourfold increase in storage for each of the three sesquiterpenes α‐bisabolol, (*E*)‐β‐caryophyllene and α‐barbatene was observed. α‐Barbatene and (*E*)‐β‐caryophyllene represent olefinic sesquiterpenes, and their physicochemical properties differ from that of α‐bisabolol, a terpene alcohol. Henry's law constants, which estimates the volatile molecules partitioning in aqueous solutions, are very different for (*E*)‐β‐caryophyllene and α‐barbatene on the one hand and α‐bisabolol on the other hand (8.1 × 10^−1^, 6.9 × 10^−1^ and 6.8 × 10^−5^ atm/m^3^/mole respectively). Formation of both lipids and sesquiterpene biosynthesis takes place in the cytoplasm, in close proximity to the endoplasmic reticulum (Pyc *et al*., 2017; Vranova *et al*., 2012). Thus, both processes seem to be spatially coupled, which may benefit the accumulation of sesquiterpenes in the neo‐synthesized lipid bodies.

Plants store their terpenes are stored in specialized tissues or cells. These include resin ducts in pine trees, glandular trichomes on mint leaves, oil cells in liverworts or idioblasts in Cinnamomum species (Glas *et al*., 2012; Li *et al*., 2016; Tanaka *et al*., 2016). In this work, we demonstrated that by inducing the accumulation of neutral lipids in leaf tissue, accumulation of essential oil can be achieved in cells which are not naturally specialized for terpene storage. Mimicking the storage of terpenes observed in specialized oil cells such as idioblasts (Kromer *et al*., 2016; Kuster and Vale, 2016). For example, idioblasts from *Arnica* species include both lipids and essential oils, where the lipids function as a medium in which the terpenes are dissolved for accumulation (Kromer *et al*., 2016). In the current study, the quantity of α‐bisabolol stored in transiently expressed heterologous lipid bodies represents only fraction of the total lipid content (~1%, w/w), which represents a concentration above 1 mm (assuming that 50% of the stored α‐bisabolol is dissolved in the lipid bodies), which leaves room for improving the ratio between lipids and terpenes. To the best of our knowledge, relative quantities of neutral lipids and sesquiterpenes in natural terpene storage cells have never been reported. Therefore, we cannot compare our findings with a natural system.

Accumulation of both neutral lipids and terpenes would theoretically be limited by the availability of acetyl‐CoA, which is a common precursor for both compound classes (Vickers *et al*., 2014; Vranova *et al*., 2013; Xu and Shanklin, 2016). Further improving the allocation balance of acetyl‐CoA to both biosynthesis pathways could improve the accumulation of stored terpenes.

### Seed lipid bodies store terpenes

The initial observation that triggered the present study was that expression of sesquiterpene synthase AtTPS21 under a 35S promoter resulted in accumulation of (*E*)‐β‐caryophyllene in seed lipid bodies, even if the 35S promoter is known to be only weakly expressed in seeds (Jefferson *et al*., 1987; Odell *et al*., 1985). Brassica seeds are known for their high oil content; for instance, Arabidopsis seeds of the Columbia ecotype, used to make the transgenic plants, have up to 35% (w/w) lipid content (Li *et al*., 2006). With this nonoptimized system, we detected a modest quantity of (*E*)‐β‐caryophyllene (~0.1 nmole per gram of dry seeds), which is much less than the quantities of sesquiterpene reported in the optimized *Camelina sativa* seeds (Augustin *et al*., 2015; Borghi and Xie, 2016). Including terpenes in lipid‐rich tissues such as seeds may prove useful in the context of bioengineering crops for the production of biofuels. Sesquiterpenes are being deployed as so‐called drop‐in molecules. For example, a molecule like (*E*)‐β‐caryophyllene has been shown to improve biofuel properties (Harvey *et al*., 2015), and expression of (*E*)‐β‐caryophyllene synthase with the appropriate promoter in the seeds of oil palm or oil seed rape could lead to improved biofuel quality.

### Expression of *RcWRI1* results in higher lipid content than *AtWRI1*

WRI1 transcription factors are important for lipid accumulation in oleaginous seeds and have been used to increase lipid accumulation in mesophyll cells of *N. benthamiana* (Grimberg *et al*., 2015). In our hands, the Arabidopsis transcription factor AtWRI1 was much less effective in driving neutral‐lipid biosynthesis than its castor bean orthologue (Figures 3 and 4). The AtWRI1 protein was shown to be subject to rapid turnover, resulting from intrinsically disordered domains and phosphorylation by the SnrK1 kinase complex (Ma *et al*., 2015; Zhai *et al*., 2017). The C‐terminal region of AtWRI1 was shown to mitigate oil accumulation upon transient expression in *N. benthamiana,* due to the presence of a PEST domain. The PEST domain is responsible for the destabilization of AtWRI1. Interestingly, analysing the WRI1 sequences using software for predicting PEST domains provided a lower score in the RcWRI1, compared to the AtWRI1 (Figure S3). Clearly, many alternative explanations of the differences between AtWRI1 and RcWRI1 are possible, including differences in expression, which are not addressed here, and it is as yet unclear whether differences in stability are at the basis of the strong difference in the ability of the two WRI1 orthologues to drive lipid body formation in *N. benthamiana*.

Contrary to the results obtained with the transcription factor WRI1, the enzyme DGAT1 cloned from Arabidopsis was more effective than the one from castor bean (Figures 3 and 4a). This result is in line with previous finding on the properties of both enzymes, which showed that RcDGAT1 is specialized in forming TAG using hydroxy‐fatty acids, which are expected to be less available in plants other than castor bean (McKeon and He, 2015). Thus, using a combinatorial approach with genes from different sources was beneficial for TAG accumulation and storage of sesquiterpenes.

## Conclusion

In this study, we demonstrate the use of the lipid bodies as a hydrophobic storage organelle for three different hydrophobic molecules from the sesquiterpene family. Improved storage was demonstrated in seeds and mesophyll cells. This work demonstrates a novel concept for engineering terpene accumulation in plants *in vivo*. Further optimizing composition and size of lipid bodies may lead to even better storage in tissues that are normally not equipped to accumulate terpenes. A system for accumulating lipid bodies and storing volatile compounds such as terpenes could also be deployed in microorganisms. Yeast, for example, which has often been used to produce plant terpenes, can also be engineered to accumulate lipid bodies, by deleting specific lipases (Kurat *et al*., 2006). It remains to be explored in how far lipid bodies can also contribute to microbial terpene accumulation. These works bring closer the possibility to produce valuable sesquiterpenes, such as α‐bisabolol, in an economically viable way in plants. This should, in the end, help to prevent overexploitation of slow‐growing plant species from vulnerable areas, as exemplified by the Candeia tree.

## Materials and methods

### Construct design

The coding sequences of castor bean (*Ricinus communis*), oil body interacting protein oleosin (name: *RcOLE1*; AY360218.1), transcription factor WRI1 (name: *RcWRI1*; NM_001323762.1), the enzyme diacylglycerol acyltransferase type‐1 (name: *RcDGAT1;* EU391591.1) were amplified from cDNA obtained from green pods. The coding sequences of *AtWRI1* (At3g54320), *AtDGAT1* (At2g19450), *AtTPS11* (At5g44630) were amplified from cDNA obtained from green pods from Arabidopsis thaliana Col0 (respectively, NM_001339659.1, NM_127503.3, NM_123830). After amplification (Table S1), the genes were cloned in a TOPO/PCR8 vector (Invitrogen), and the orientation and sequences were confirmed by Sanger sequencing. Subsequently, the coding sequences were introduced in a binary vector (pB7GW2.0) under control of a 35S constitutive promoter. The α‐bisabolol‐synthase from *Artemisia annua* (*AaBOS*) used for this project was obtained synthetically (Genscript), based on the gene bank sequence AFV40969. The *AtTPS21* construct used in this study was previously described in Ting *et al*. (2015). The constructs were transferred to *Agrobacterium tumefaciens* strain AGL0 by electroporation.

### Agro‐infiltration

Agro‐infiltration experiments were performed as described in van Herpen *et al*. (2010). In brief, *Agrobacterium* strains were grown at 28°C at 220 rpm for 48 h in LB media with spectinomycin/rifampicin (100 mg/L). Cells were collected by centrifugation for 20 min at 2000 ***g*** (15°C) then resuspended (10 mm MES, 10 mm MgCl_2_ and 100 μm 4′‐hydroxy‐3′,5′‐dimethoxyacetophenone; Sigma) to a final OD_600_ of 0.5, followed by incubation for 2 h (50 rpm, RT). For co‐infiltration, equal volumes of the *Agrobacterium* strains were mixed. In all experiments, an *Agrobacterium* strain harbouring a gene encoding the TBSV P19 protein was added to suppress gene silencing (Qu and Morris, 2002). *N. benthamiana* plants were grown from seeds on soil in a greenhouse with 16‐h light at 28°C (16 h)/25°C (8 h). Strain mixtures were infiltrated into leaves of 4‐week‐old *N. benthamiana* plants using a 1‐mL syringe. The plants were grown under greenhouse conditions until further analysis.

Arabidopsis (Columbia‐0) stable transformants were obtained by floral dipping using *Agrobacterium* containing the 35S:AtTPS21 plasmid (Liu, 2011). Primary transformants were screed in vitro on (&frac12; MS 1% agar) plates containing 50 μg/mL kanamycin, and selected transformed lines were made homozygous. The production of (*E*)‐β‐caryophyllene was confirmed in Arabidopsis as described latter for *N*. *benthamian*a.

### Lipid measurement

Total lipid quantification adapted with minor modifications from Vanhercke *et al*. (2014). Leaf samples were collected, snap‐frozen in liquid N_2_ and freeze‐dried overnight. After weighing, the leaves were ground with mortar and pestle and mixed with chloroform: methanol: acetone 2:1:0.5 (v/v, 6 mL). Samples were collected in glass tubes which were sonicated 15 min (sonicator Branson 3510) and vortexed after adding 2 mL of 0.1 M KCl. The lower phase (chloroform: methanol, 1 mL) below the layer of leaf debris was collected after centrifugation for 10 min at 5000 ***g*** (centrifuge Harrier 15/80 MSE). The remaining part was washed with 1 mL of chloroform and centrifuged at 5000 ***g*** for 10 min, and then, the lipid phase was collected again (1 mL) and merged with the previous one. An aliquot of 100 μL was also collected for GC‐MS analysis. Solvent was evaporated under N_2_ flow and the weight of the samples measured to determine gravimetrically the quantity of total lipids. The dried samples were then dissolved in a known volume of chloroform per mg of leaf dry weight (5× dry weight) for TLC analysis.

### Thin‐layer chromatography

Plates (silica gel TLC plates, 60 Å Al foils with fluorescent indicator 254 nm, 20 × 20 cm – Sigma‐Aldrich, used with dimensions of 20 × 10 cm) were activated at 100°C for 45 min after a prewash in chloroform: methanol 2 : 1 (v/v) and methanol: water 1 : 1 (v/v). The samples diluted in methanol (5× DW) were applied with glass pipettes. TLC separation was performed in a closed glass chamber containing a solution of hexane: diethylether: acetic acid 90 : 20 : 1.5 (v/v/v), according to Fowler *et al*. (1987). The separation was carried out until the solvent reached 1 cm from the top, and then, plates were stabilized by drying in oven at 100°C for 5 min. The plates were developed with iodine vapour. A TAG calibration curve was constructed with a commercially available extra‐virgin olive oil diluted in chloroform. 0.5 g of oil was diluted in 1 mL of chloroform and used as first sample; this was then diluted in chloroform from 10× to 1,000,000×. The developed TLC plates were imaged using a table top scanner (EPSON v330 Photo), and images were analysed and lipid spots quantified with ImageJ (version 1.4 g) software (Schneider *et al*., 2012).

### GC‐MS analysis

Sesquiterpene quantification was performed with GC‐MS. The chloroform: methanol: acetone 2:1:0.5 (v/v/v) extracts were dried by passing them through a Pasteur pipette filled with anhydrous sodium sulphate (Sigma‐Aldrich). Samples were analysed on an Agilent 7890A gas chromatograph connected to a 5795C mass selective Triple‐Axis Detector (Agilent Technologies, United States). For that purpose, 1 μL of extract was injected at 250 °C in splitless mode on a ZB‐5MS column (Phenomenex, 30 m × 0.25 mm; ID 0.25 μm) with 5 m guard column with a constant flow of helium at 1 mL/min. The oven was programmed for 1 min at 45 °C and then subsequently ramped at 10 °C/min to 300 °C and kept for a final time of 5 min with a solvent delay of 5.5 min. The ionization potential was set at 70 eV, and scanning was performed from 45 to 400 atomic mass units, with a scanning speed of 3.99 scans/sec. Quantification was performed with an external calibration curve from α‐bisabolol (Sigma‐Aldrich). The Henry's law constants were calculated with the HENRYWIN program available online (https://www.epa.gov/tsca-screening-tools/epi-suitetm-estimation-program-interface) at 25 °C [HENRYWIN v3.10] Bond Method.

### Light microscopy

Transformed leaves were subjected to destaining overnight in 70% ethanol, followed by staining in Sudan IV (Sigma‐Aldrich) solution for 30 min (0.3 g of Sudan IV powder in 10 mL of 70% ethanol). After Sudan IV treatment, destaining was performed in 70% ethanol and then in water. Samples stained with NADI were placed for 30 min in a freshly prepared solution of 1‐naphtol:N,N‐dimethyl‐p‐phenylenediamine dihydrochloride: ethanol (w/v, 1‰:1‰:40%) in 10 mm phosphate buffer (pH 7.2) and then washed for 2 min in 100 mm phosphate buffer (pH 7.2). Samples were covered with a drop of glycerine (80%, Sigma‐Aldrich). All images were collected on a Nikon optiphot‐2 (Nikon) equipped with a Axiocam camera (ERc 5s, Zeiss). Lipid bodies were measured with the ImageJ software. The average size of lipid bodies observed in the focal plan of the *RcO* + *AtDGAT1* (103 lipid bodies from eight biological replicates) and *RcO *+ *AtDGAT1 *+* RcWRI1* (183 lipid bodies from 10 biological replicates) samples was quantified using the ImageJ software.

### Data analysis

Statistical differences between two samples were performed with a Student's *t*‐test in Excel (Microsoft Office Professional 2016). The experiments were designed with at least three replicates.

## Conflict of interest

The authors affirm that they do not have any associated conflict of interest.

## Supporting information


**Figure S1** TLC plates with iodine staining (black and white filter) of leaf extract samples at 7 DPI: (a) empty vector, (b) RcO1 + RcDGAT1, (c) RcO + AtDGAT1, (d) RcO + AtDGAT1 + RcWRI1, (e) RcO+RcDGAT1 + RcWRI1.
**Figure S2** Mesophyll cells by light microscopy.
**Figure S3** PEST score estimation. Using the PEST domain prediction tool EpestFind (http://emboss.bioinformatics.nl/cgi-bin/emboss/epestfind) for AtWRI1 and RcWRI1.
**Table S1** primers used in this study.
