# Supplementary material for: Engineering storage capacity for volatile sesquiterpenes in Nicotiana benthamiana leaves
Source: Plant Biotechnol J. 2018 May 28;16(12):1997–2006. doi: 10.1111/pbi.12933 (PMC6230952; doi:10.1111/pbi.12933)
Supplement: Supplementary file 1 — Figure S1 TLC plates with iodine staining (black and white filter) of leaf extract samples at 7 DPI: (a) empty vector, (b) RcO1 + RcDGAT1, (c) RcO + AtDGAT1, (d) RcO + AtDGAT1 + RcWRI1, (e) RcO+RcDGAT1 + RcWRI1. Figure S2 Mesophyll cells by light microscopy. Figure S3 PEST score estimation. Using the PEST domain prediction tool EpestFind (http://emboss.bioinformatics.nl/cgi-bin/emboss/epestfind) for AtWRI1 and RcWRI1. Table S1 primers used in this study. [file PBI-16-1997-s001.pptx]

## Slide 1
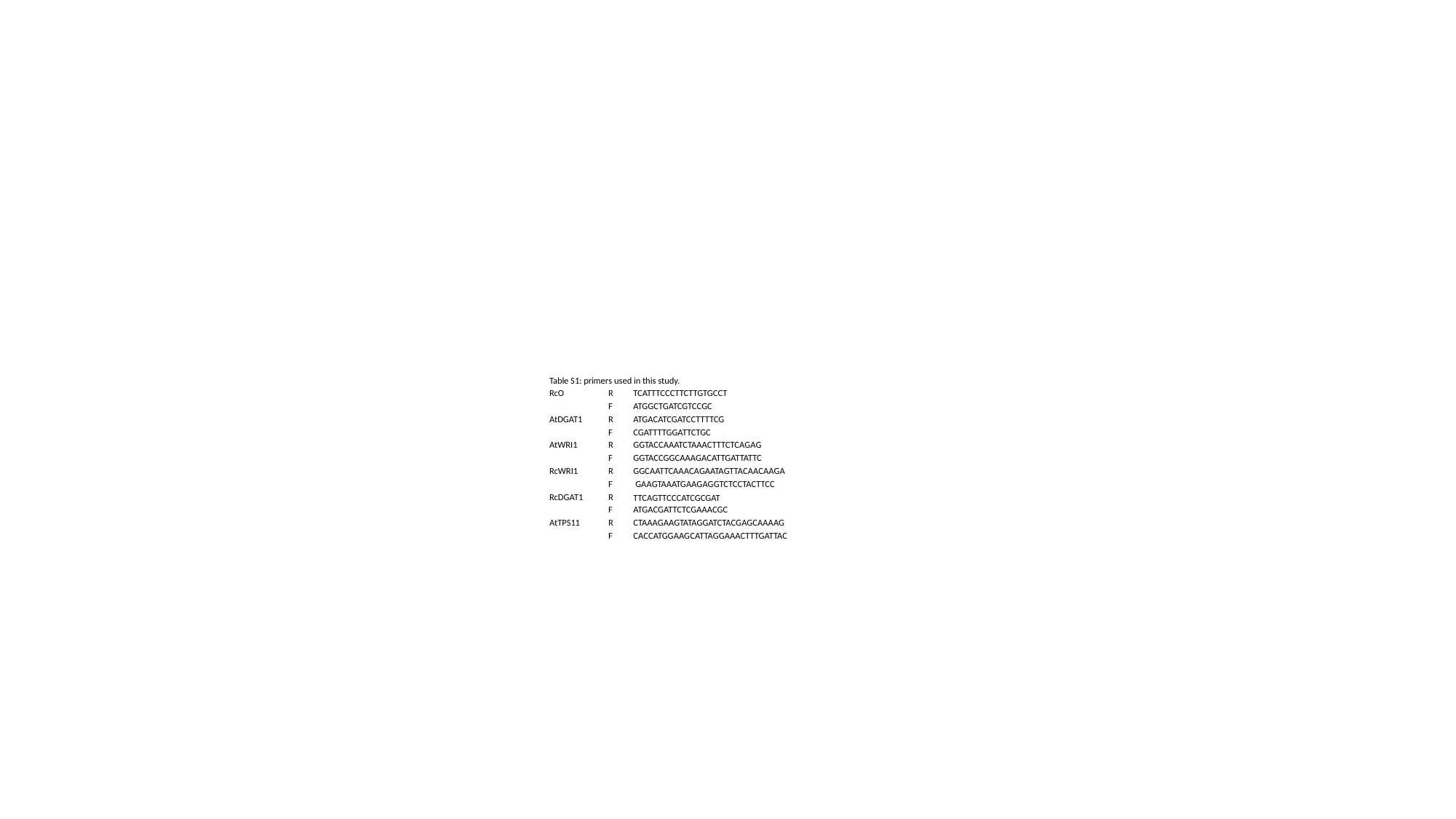

| Table S1: primers used in this study. | | |
| --- | --- | --- |
| RcO | R | TCATTTCCCTTCTTGTGCCT |
| | F | ATGGCTGATCGTCCGC |
| AtDGAT1 | R | ATGACATCGATCCTTTTCG |
| | F | CGATTTTGGATTCTGC |
| AtWRI1 | R | GGTACCAAATCTAAACTTTCTCAGAG |
| | F | GGTACCGGCAAAGACATTGATTATTC |
| RcWRI1 | R | GGCAATTCAAACAGAATAGTTACAACAAGA |
| | F | GAAGTAAATGAAGAGGTCTCCTACTTCC |
| RcDGAT1 | R | TTCAGTTCCCATCGCGAT |
| | F | ATGACGATTCTCGAAACGC |
| AtTPS11 | R | CTAAAGAAGTATAGGATCTACGAGCAAAAG |
| | F | CACCATGGAAGCATTAGGAAACTTTGATTAC |

## Slide 2
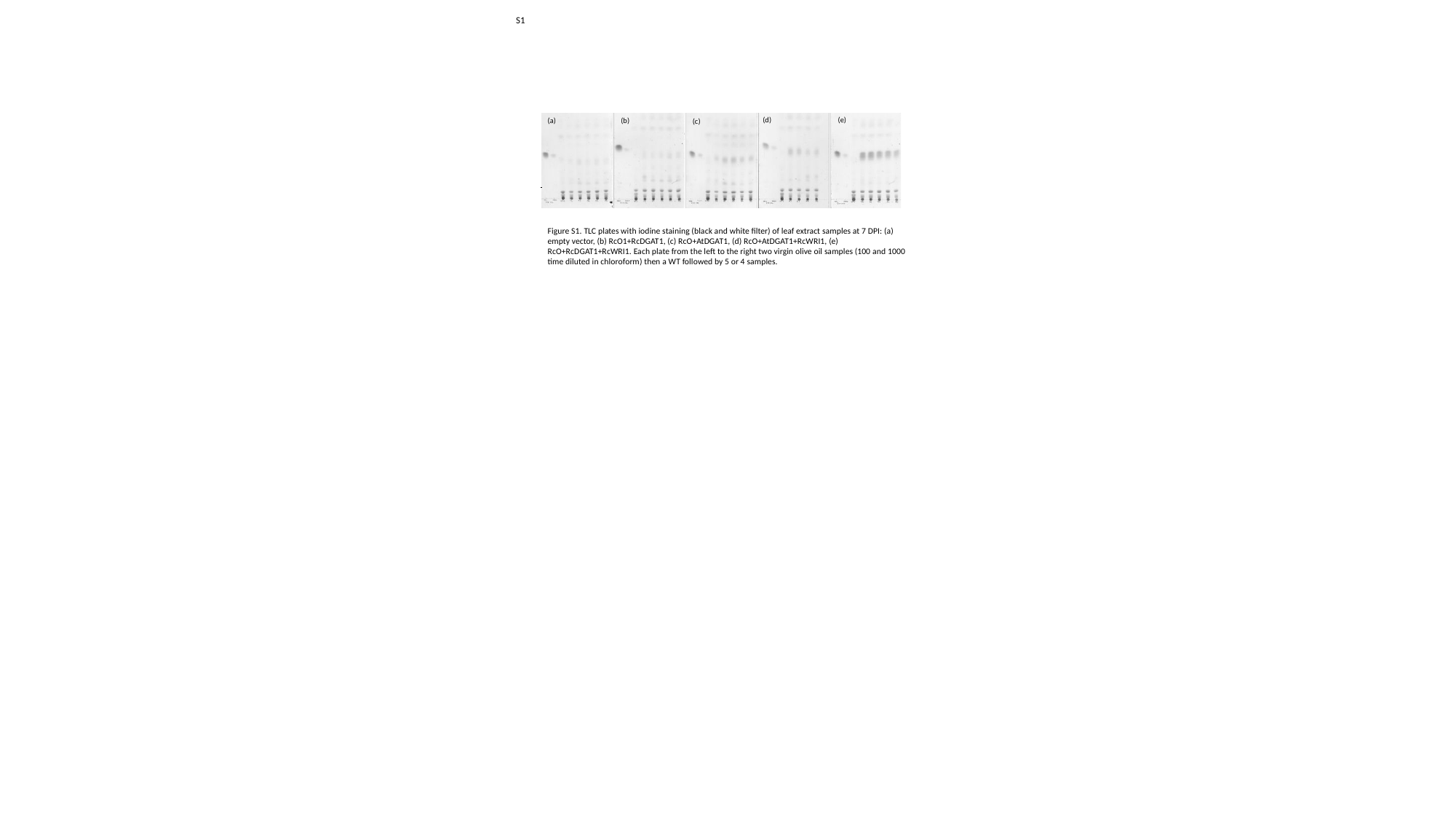

S1
(e)
(d)
(a)
(b)
(c)
Figure S1. TLC plates with iodine staining (black and white filter) of leaf extract samples at 7 DPI: (a) empty vector, (b) RcO1+RcDGAT1, (c) RcO+AtDGAT1, (d) RcO+AtDGAT1+RcWRI1, (e) RcO+RcDGAT1+RcWRI1. Each plate from the left to the right two virgin olive oil samples (100 and 1000 time diluted in chloroform) then a WT followed by 5 or 4 samples.

## Slide 3
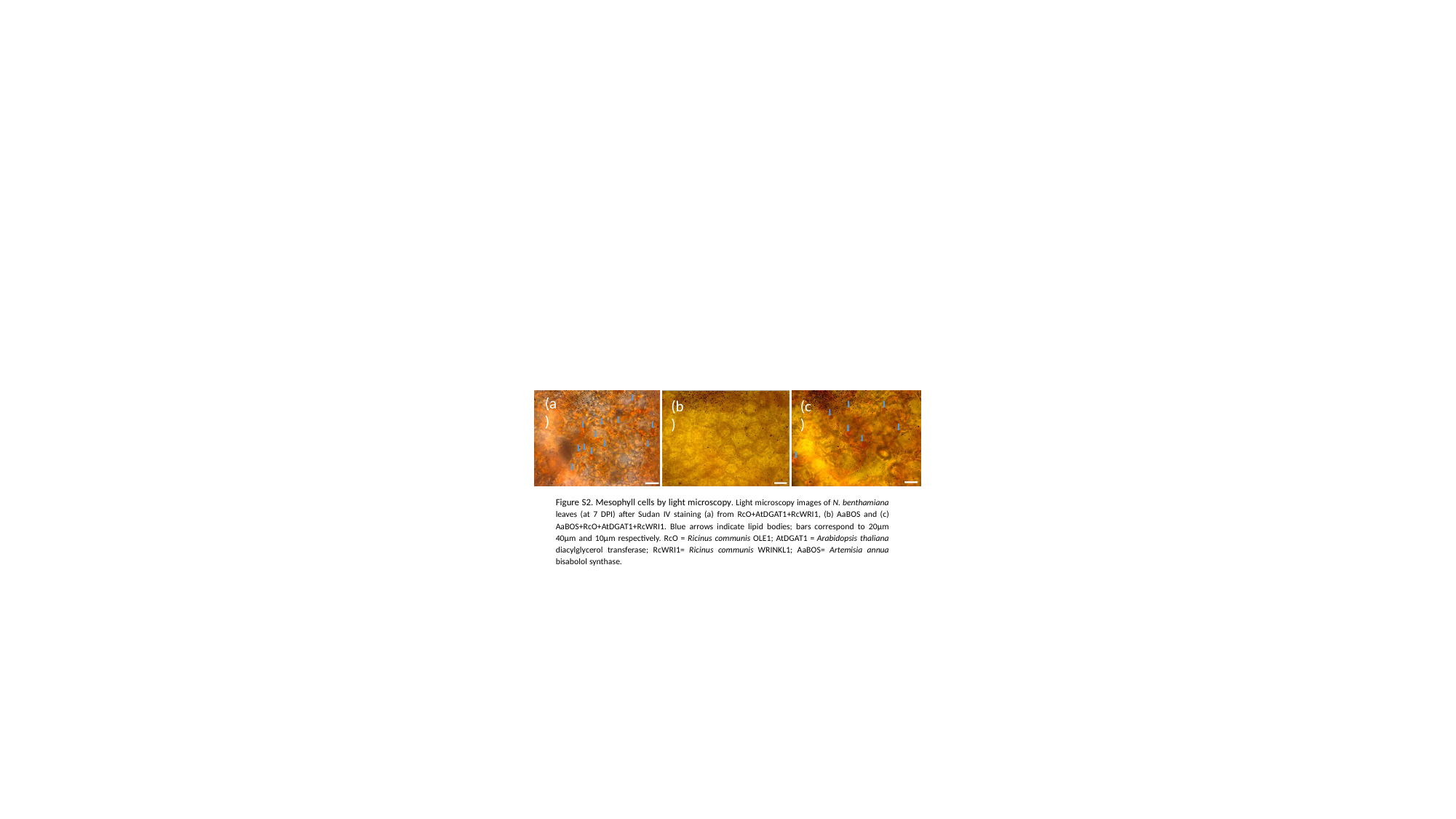

(a)
(b)
(c)
Figure S2. Mesophyll cells by light microscopy. Light microscopy images of N. benthamiana leaves (at 7 DPI) after Sudan IV staining (a) from RcO+AtDGAT1+RcWRI1, (b) AaBOS and (c) AaBOS+RcO+AtDGAT1+RcWRI1. Blue arrows indicate lipid bodies; bars correspond to 20µm 40µm and 10µm respectively. RcO = Ricinus communis OLE1; AtDGAT1 = Arabidopsis thaliana diacylglycerol transferase; RcWRI1= Ricinus communis WRINKL1; AaBOS= Artemisia annua bisabolol synthase.

## Slide 4
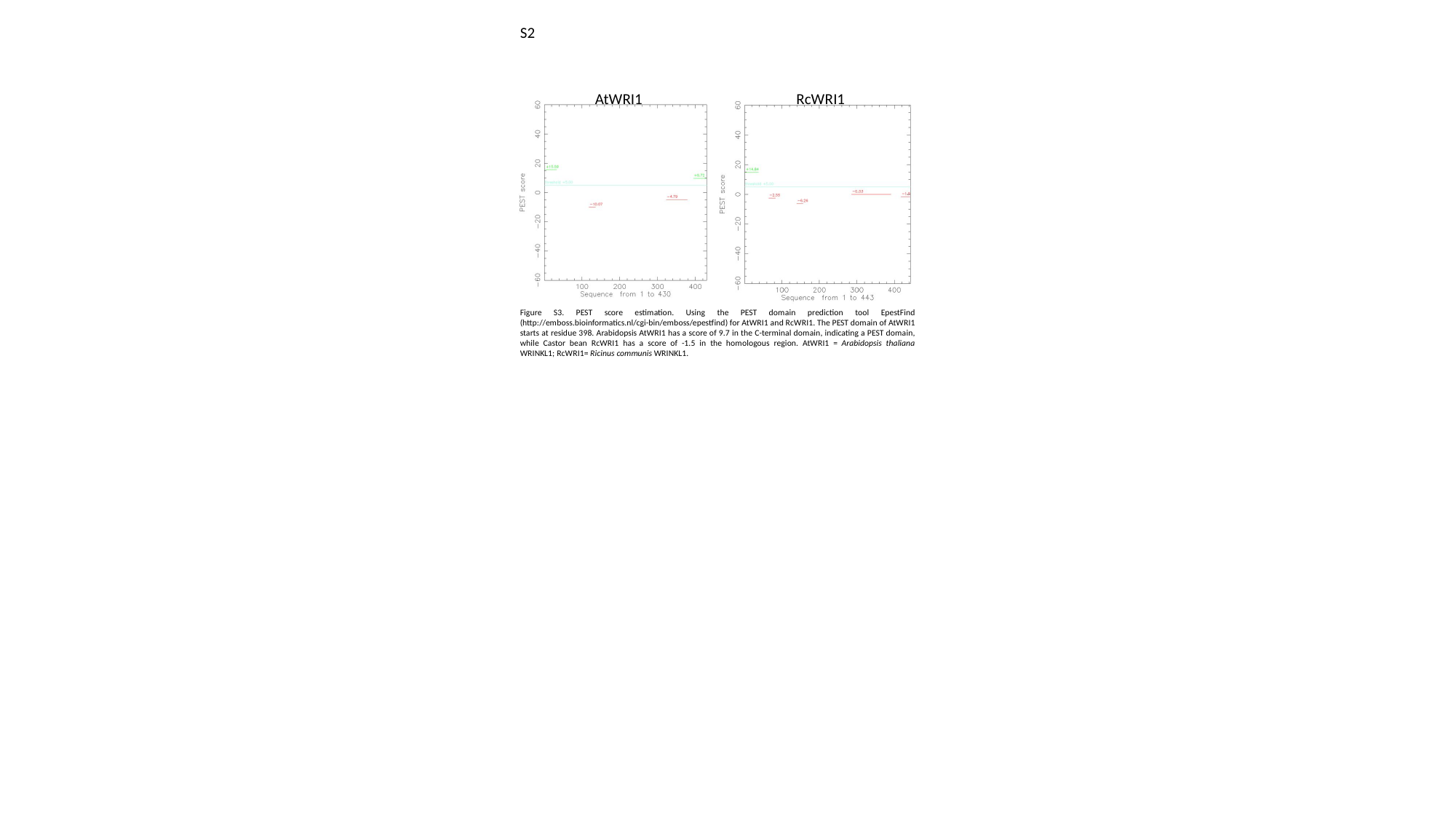

S2
AtWRI1
RcWRI1
Figure S3. PEST score estimation. Using the PEST domain prediction tool EpestFind (http://emboss.bioinformatics.nl/cgi-bin/emboss/epestfind) for AtWRI1 and RcWRI1. The PEST domain of AtWRI1 starts at residue 398. Arabidopsis AtWRI1 has a score of 9.7 in the C-terminal domain, indicating a PEST domain, while Castor bean RcWRI1 has a score of -1.5 in the homologous region. AtWRI1 = Arabidopsis thaliana WRINKL1; RcWRI1= Ricinus communis WRINKL1.
